# A novel mechanism for variable phenotypic expressivity in Mendelian diseases uncovered by an AU-rich element (ARE)-creating mutation

**DOI:** 10.1186/s13059-017-1274-3

**Published:** 2017-07-28

**Authors:** Nisha Patel, Arif O. Khan, Maher Al-Saif, Walid N. Moghrabi, Balsam M. AlMaarik, Niema Ibrahim, Firdous Abdulwahab, Mais Hashem, Tarfa Alshidi, Eman Alobeid, Rana A. Alomar, Saad Al-Harbi, Mohamed Abouelhoda, Khalid S. A. Khabar, Fowzan S. Alkuraya

**Affiliations:** 1 Department of Genetics, King Faisal Specialist Hospital and Research Center, Riyadh, Saudi Arabia; 2 Eye Institute, Cleveland Clinic Abu Dhabi, Abu Dhabi, 112412 United Arab Emirates; 3 Program in BioMolecular Research, King Faisal Specialist Hospital and Research Center, Riyadh, Saudi Arabia; 4 King Khaled Eye Specialist Hospital, Riyadh, Saudi Arabia; 5 Department of Anatomy and Cell Biology, College of Medicine, Alfaisal University, Riyadh, Saudi Arabia; 6 Saudi Human Genome Program, King Abdulaziz City for Science and Technology, Riyadh, Saudi Arabia

**Keywords:** AU-rich elements, Variable expressivity, Tissue-specific, Cornea, 3′UTR

## Abstract

**Background:**

Variable expressivity is a well-known phenomenon in which patients with mutations in one gene display varying degrees of clinical severity, potentially displaying only subsets of the clinical manifestations associated with the multisystem disorder linked to the gene. This remains an incompletely understood phenomenon with proposed mechanisms ranging from allele-specific to stochastic.

**Results:**

We report three consanguineous families in which an isolated ocular phenotype is linked to a novel 3′ UTR mutation in SLC4A4, a gene known to be mutated in a syndromic form of intellectual disability with renal and ocular involvement. Although SLC4A4 is normally devoid of AU-rich elements (AREs), a 3′ UTR motif that mediates post-transcriptional control of a subset of genes, the mutation we describe creates a functional ARE. We observe a marked reduction in the transcript level of SLC4A4 in patient cells. Experimental confirmation of the ARE-creating mutation is shown using a post-transcriptional reporter system that reveals consistent reduction in the mRNA-half life and reporter activity. Moreover, the neo-ARE binds and responds to the zinc finger protein ZFP36/TTP, an ARE-mRNA decay-promoting protein.

**Conclusions:**

This novel mutational mechanism for a Mendelian disease expands the potential mechanisms that underlie variable phenotypic expressivity in humans to also include 3′ UTR mutations with tissue-specific pathology.

## Background

All the cells of an individual share an identical genome, barring postzygotic mutations, yet they display strikingly different structural and functional characteristics attributed to diverse transcriptional profiles [1]. This basic concept is frequently invoked to explain the selective involvement of a subset of organs in diseases caused by germline mutations, i.e. only tissues that express the mutated gene are subject to the pathological effect of mutations therein. However, the observation that in many pleiotropic diseases, the multisystem involvement is highly variable among patients (also known as variable expressivity), poses a challenge to this concept since the gene is clearly both expressed and required by the tissues that are only affected in some patients but not others [2]. Variable expressivity is one of the most challenging obstacles in variant interpretation and understanding its underlying mechanism, therefore, can have a significant impact on the delivery of genomic medicine.

Several mechanisms have been proposed to explain variable expressivity and they need not be mutually exclusive [3]. For example, Bardet–Biedl syndrome is a multisystem ciliopathy and yet several mutations have been reported to cause an eye-limited phenotype. At least in one such instance, it has been shown that the eye-specific mutation affects an eye-specific transcript, consistent with the identification of tissue-specific exons [4, 5]. Allele-specific variable phenotypic expressivity can also uncover a tissue-variable threshold where tissues display different susceptibility depending on the level of gene deficiency. For example, mild mutations in the gene encoding phosphoglycerate dehydrogenase cause a brain-specific phenotype whereas more severe deficiency causes a lethal multisystem disease known as Neu-Laxova syndrome [6, 7]. Alternatively, coding mutations may cause a pleiotropic phenotype whereas enhancer mutations will only affect the tissues in which that enhancer is active, as observed in *PTF1A*-related cerebellar and pancreatic agenesis versus *PTF1A*-related isolated pancreatic agenesis [8, 9]. However, this genotype/phenotype correlation is often lacking and variable expressivity can be seen in the context of the same mutation. Proposed mechanisms in such instances include variable splicing efficiency (e.g. *TCTN1*-related ciliopathies), the requirement for postzygotic second mutations (e.g. neurofibromatosis), or other genetic modifiers, as well as stochastic events [10, 11]. Clearly, much remains to be understood about the factors that control this phenomenon.

Mutations in the untranslated region (UTR) of genes are extremely rare causes of Mendelian diseases and even the few described (e.g. *FMR1*-related fragile-X syndrome, *DMPK*-related myotonic dystrophy) tend to represent repeat expansion. While 5′UTR point mutations causing Mendelian diseases have rarely been described, 3′UTR mutations in the context of Mendelian phenotypes are even rarer [12–15]. A few of these examples include a compound heterozygous mutation in GFPT1, which includes a 3′UTR mutation (c.*22C > A) has been reported to cause congenital myasthenic syndromes by the formation of a microRNA target site [16]. Additionally, 3′UTR mutation in FMR1 has been shown to cause fragile X syndrome by disrupting the mRNA binding protein HuR, leading to destabilization and rapid degradation of the *FMR1* transcript [17]. Interestingly, both 5′UTR and 3′UTR mutations in GJB1 have been reported to cause Charcot–Marie–Tooth disease [18]. In the context of multifactorial diseases, however, several 3′UTR SNPs have been reported with suggested influence on disease susceptibility [19]. These include rs356165 in *SNCA* encoding alpha-synuclein as a potential susceptibility locus for Parkinson disease [20], rs3027898 in *IRAK1* as a potential susceptibility locus for rheumatoid arthritis [21], and another 3′UTR SNP in *hPTP1B* associates with insulin resistance [22]. In this study, we describe, to our knowledge, the first ARE-creating mutation in the context of a Mendelian phenotype and propose a novel allele-specific mechanism for variable expressivity as a result.

## Methods

### Human participants

Patients were evaluated clinically followed by enrollment in an IRB-approved research protocol with informed consent (KFSRHC RAC# 2070023). Parents and available siblings were also recruited. Venous blood was collected from all participants in EDTA tubes and, in a subset of patients, in sodium heparin tubes for DNA extraction and establishment of lymphoblastoid cell lines (LCL), respectively.

### Autozygosity mapping and exome sequencing

DNA samples from all available family members were genotyped on the Axiom SNP chip platform following the manufacturer’s instructions, followed by autozygome analysis using AutoSNPa [23]. Runs of homozygosity that are > 2 Mb in length were considered as surrogates of autozygosity given the consanguineous nature of the families and the overlapping autozygome between the affected individuals only was considered as a candidate positional locus as described before [24, 25]. For exome analysis, we selected the index from two families. The samples were prepared according to the preparation guide of Agilent SureSelect Target Enrichment Kit and the resulting libraries were sequenced using the Illumina HiSeq2000 sequencer. The Genome Analysis Toolkit (GATK) was used for variant calling.

### mRNA level assays

In the two human participants for which LCL were established, RNA was extracted from cell pellets followed by relative quantification of the SLC4A4 (MIM#604278). Total RNA from patients and control lymphoblast cell lines were extracted using QIAamp RNA mini Kit (Qiagen inc., Germantown, MD, USA) with DNAase treatment (Qiagen), according to manufacturer’s instructions. cDNA was prepared using the iScript™ cDNA synthesis kit and poly-T oligonucleotide primers (Applied Biosystems, Carlsbad, CA, USA). Relative quantification reverse transcription polymerase chain reaction (RT-QPCR) was performed using SYBR green and Applied Biosystems 7500 Fast Real-Time PCR system. Primers were designed to flank an intron to specifically amplify cDNA (*SLC4A4* 5’- ATTCCTTGAACGCCACACAT; 5’- TTTCTGTTCCCTTGCTCCTC), generating 159 bp amplicon. Melt curve analysis was performed to confirm that a single product was amplified. Gene expression was normalized to *GAPDH* and all reactions were run in triplicate and repeated as three independent experiments.

### Cells

HeLa epithelial cells, human kidney HEK293 cell line, neuroblastoma NB-A, and normal human fibroblasts were obtained from the American Type Culture Collection (ATCC; Rockville, MD, USA). The HAP-1 wild-type (WT) and CRISPR ZFP36-knockout haploid fibroblast –like cell line were obtained from Horizon Discovery (UK) and cultured in Iscove’s Modified Dulbecco’s Medium with 10% fetal bovine serum (FBS) and 1% antibiotics. HeLa and HEK cell lines were maintained in Dulbecco’s modified Eagle’s medium (DMEM), while neutroblastoma and fibroblasts were maintained in MEM (ThermoFisher, Carlsbad, CA, USA) supplemented with 10% (FBS) and antibiotics. Tet-On and Tet-Off Advanced HeLa cells were obtained from ClonTech and were maintained in DMEM supplemented with 10% Tet-System Approved FBS (ClonTech) and antibiotics and maintained in selection medium (100 μg/mL G418; Sigma).

### Cloning of ARE reporter constructs

The RPS30 promoter (RPS30)-linked reporter expression vectors [26] containing a 70-base region from the normal control *SCL4A4* 3′UTR and the ARE-creating mutant 3′UTR were constructed using synthetic oligos. The oligo sequence is: TGTCATTTGTTTTTGTTTGGCTGTTT***G***TTTATTTTTTAACTTTTATTTCGTCTCAGTTT TTGG, where G to A is in the oligo with the ARE-creating mutant. The oligonucleotides were designed to contain (G/GATCC) BamHI and (T/CTAGA) XbaI overhangs. The annealed oligos were cloned in BamH1 and XBaI sites in the 3′UTR of the RPS30-nanoluciferase vector. The use of the transcriptionally non-inducible RPS30 promoter allows selective post-transcriptional effects.

### Transfection and reporter activity measurements

Cells were plated at 2 × 10^5^ cells/mL per well in 96-well plate overnight and co-transfected with RPS30 promoter-linked nanoluciferase fused with the 3′UTR containing WT and mutant sequences along with firefly luciferase vector as a normalization control. The cells were transfected using Lipofectamine 2000 (Invitrogen) according to the manufacturer’s instructions. Transfections were performed in several replicates. After 16 h, cells were lysed and assayed for luciferase activity using the dual Nano-Glo dual-luciferase assay kit (Promega) according to the manufacturer’s instructions and measured on a luciferase luminometer. Data were presented as mean ± SEM of normalized Nano luciferase intensity/firefly intensity.

### mRNA half-life experiments

For actinomycin D chase-based mRNA half-life determination, actinomycin D (5 μg/mL; Sigma) was used to block transcription. For Tet-off based determination of mRNA half-life, the reporter constructs were converted to Tet-regulated cassettes by inserting multiple copies of TetO sites in a minimal cytomegalovirus (CMV) promoter, as previously described [27]. The Tet-off reporters fused with the WT or mutant 3′UTR were transfected to HeLa Tet-off cells overnight and then treated with doxycycline (0.25 μg/mL) to shut off transcription for 1, 2, 4, 6, and 24 h.

The RNA was extracted by Trizol at multiple points following transcriptional shut off and then subjected to RT-QPCR. The RT reaction was performed using total RNA, 150 ng random primers, 10 mM dNTP mixture, 40 U/μL RNase inhibitor, and 200 units of SuperScript II RT. RT-QPCR was performed as multiplex reactions in a CFX96 cycler (Bio-Rad, Hercules, CA, USA) using FAM-labeled TaqMan probe/primers for Nanoluciferase: the nanoluciferase primer/probes were custom-synthesized by Metabion: (forward primer: 5′-CTCCATCTTCGCGGTAGCT -3′, reverse primer: 5′-GAGGACTTGGTCCAGGTTGTA -3′, and FAM-labeled TaqMan probe: 5′-Fam-CCGCCGTTCAGTCGCCGT -BHQ-1-3′). The RNA samples were normalized with VIC-labeled ribosomal protein (PO) probe. Samples were amplified in triplicate and quantification of relative expression was performed using the ΔΔCt method. The mRNA half-life was calculated using the one-phase exponential decay method (GraphPad Software, San Diego, CA, USA). The equation Y = Span × exp(–K × X) + Plateau describes the kinetics of mRNA decay. X is time and Y represents the response. SPAN and PLATEAU are expressed in the same units as the Y axis. K is expressed in the inverse of the units used by the X axis. Y starts as equal to SPAN + PLATEAU and decreases to PLATEAU with a rate constant K. The mRNA half-life of the decay is 0.6932/K. This method has the best fit for mRNA data that tend to decay at a certain rate over a period of time and then reach a plateau. Using the least squares fit method, the fits were > R = 0.9.

### RNA immunoprecipitation and immunoblotting

HEK-293 cells were transfected overnight with 500 ng vector expressing HA-tagged ZFP36 (previously known as TTP). Cells were lysed in RNA immunoprecipitation (RNA-IP) buffer consisting of 0.5% NP40, 100 mM KCl, 10 mM HEPES (pH 7.0), 5 mM MgCl_2_, freshly supplemented before use with 1 mM DTT, 5 μL/mL units RNase Out (ThermoFisher), and protease inhibitor mix (Roche). The lysates were centrifuged at 12,000 rpm and the supernatants obtained were incubated with monoclonal anti-HA antibody (for ZFP36) or monoclonal anti-Myc antibody as background control (coupled with pre-swollen protein G-sepharose beads). Aliquots were collected for western blotting and the remaining beads were used for RNA extraction using RNazol (Sigma), followed by chloroform and isopropanol precipitation. The cDNA was synthesized using SuperScript II RT (ThermoFisher). RT-QPCR was performed in multiplex reaction in a thermal cycler (Bio-Rad) using the nanoluciferase and PO primer/probe combination.

To confirm transfection and expression of the ZFP36, total lysates were subjected to western blotting using anti-HA antibody while anti-Myc antibody was used as a negative control as previously described [28]. Signal detection was performed with ECL western blotting detection reagents (Amersham Biosciences, UK). Protein molecular weight markers were used to verify the protein size.

### Functional response of the *SLC4A4* neo-ARE towards ZFP36

Tetracycline inducible (Tet-On) ZFP36 expressing cassettes were constructed by PCR of pCR3.1-ZFP36 plasmids using the forward primer that includes multiple copies of the TetO site as previously described [29]. HeLa Tet-On Advanced cells were transfected with 50 ng of either the WT or mutant 3′UTR fused-nanoluiferase reporters along with Firefly plasmid normalization control and 10 ng of the TetO-ZFP36 constructs. Transfections were performed using Lipofectinamine 2000 (Invitrogen) according to the manufacturer’s instructions. Doxycycline (0.25 ug/mL) was added to the transfected cells for 16 h and luminescence was acquired by luminometer. Data were presented as mean ± SEM of normalized Nano luciferase intensity/firefly intensity.

## Results

### Isolated keratopathy is caused by a 3′UTR variant in *SLC4A4*

**Fig. 1:**
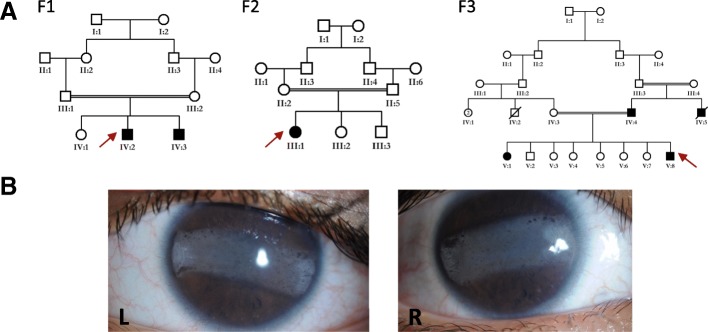
Clinical images and family pedigree of three families with isolated band keratopathy. **a** Pedigree of three families. *Red arrows* indicate the proband in each family. **b** *Photograph* of individual F1:V2 shows band keratopathy in both the right and left eye

Three consanguineous Saudi families that share the same tribe name but are not known to be directly related were identified through clinical evaluation for impaired vision in an ophthalmology clinic for a total of six affected members (Fig. 1). None had history of developmental delay or renal disease. Physical examination revealed normal growth parameters. Family 1: two brothers with bilateral progressive band keratopathy first noted at approximately two years of age. Ophthalmic evaluation was otherwise unremarkable. There was no evidence of metabolic, kidney, or liver disease in either of the two affected brothers. Family 2: a boy with bilateral progressive band keratopathy first noted at approximately three years of age. He was also noted to have increased intraocular pressure. At the time of evaluation there was no evidence of metabolic, kidney, or liver disease. Family 3: a brother and sister with congenital glaucoma who underwent glaucoma surgery soon after birth and were noted to develop band keratopathy at around two years of age.

**Fig. 2:**
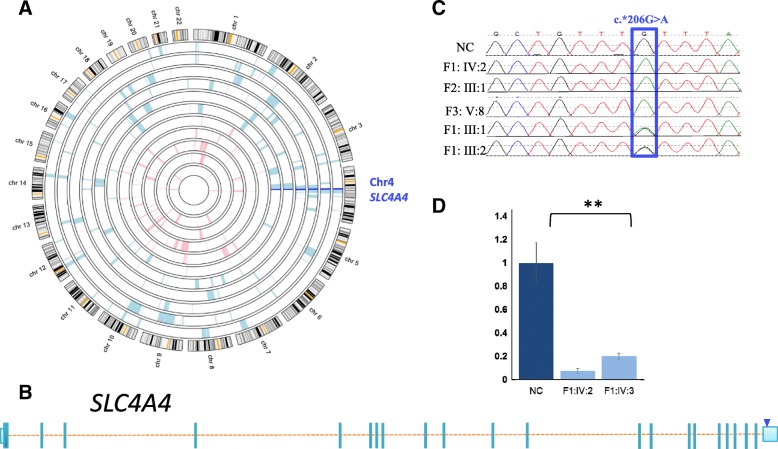
3′UTR mutation in *SLC4A4* is discovered in three families. **a** Agile Multi Ideogram showing the exclusive region of homozygosity (ROH) between the affected individuals in the three families (*dark blue*) on Chr4 that is not shared with any of the unaffected individuals (*pink*). *Light blue* and *pink blocks* denote ROHs present in affected and unaffected members, respectively. **b** *Schematic* of *SLC4A4* gene that is 233.03Kb in length. *Small blue box* indicates UTR region, while the *triangle* locates the position of the mutation identified by whole-exome sequencing. **c** Genomic DNA sequence chromatogram of the 3′UTR mutation that was found to segregate in all three families. **d** Analysis of *SLC4A4* expression by RT-QPCR in two patient LCL compared to two gender matched controls reveals > 85% reduction in expression of the gene. Results are normalized to *GAPDH* and are an average from triplicate readings from three independent experiments. *P* values are paired Student’s t-test, **P < 0.01. *Error bars* are SEM

Autozygosity mapping pointed to a single shared haplotype on Chr4 that is exclusively shared by the affected members of the three families (Chr4:70893750-77748290). Exome sequencing of one index from family 1 and family 3 revealed a single shared novel homozygous variant between the two sequenced individuals. This variant NM_003759.3:c.*206G>A is in the 3′UTR of *SLC4A4* (Fig. 2), a gene known to be mutated in a rare autosomal syndromic form of intellectual disability that is characterized by severe renal tubular acidosis and ocular involvement, the latter being in the form of glaucoma and keratopathy. This variant was confirmed by Sanger sequencing and segregated with the phenotype in all three family with the members available and was absent in ExAC and GnomAd (accessed June 5, 2017) [30]. Although all genes within the critical locus received adequate coverage, we performed RT-PCR to exclude the possibility of a deep intronic mutation in SLC4A4 that is in linkage disequilibrium with the 3’UTR variant; there was no evidence of aberrant splicing (data not shown).

### Isolated band keratopathy mutation creates an AU-rich element

**Fig. 3:**
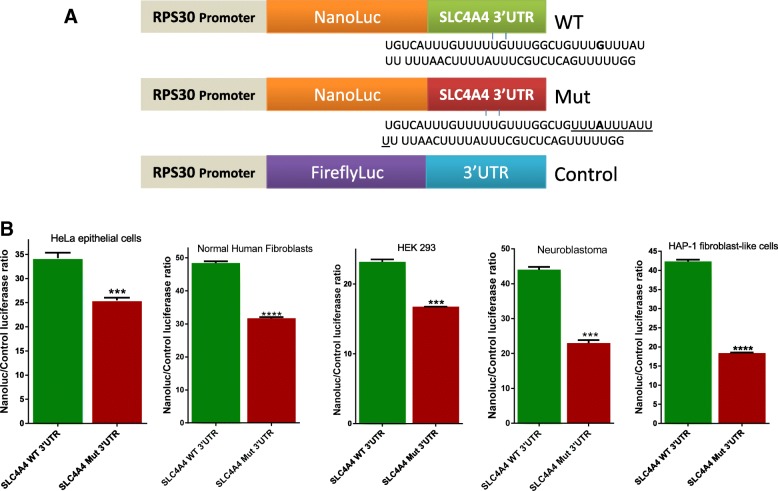
ARE-forming mutation of *SLC4A4* and its effects on post-transcriptional regulation. **a** A scheme showing the ARE-forming mutation (*underlined*) as a result of the G to A substitution and constructs used in the study. **b** Several cell types, as indicated, were co-transfected with nanoluciferase (NanoLuc) reporter fused with WT *SLC4A4* 3′UTR or the ARE-forming mutant -*SLC4A4* 3′UTR together with control firefly luciferase expression vector, for 16 h. Cells were lysed and luciferase activity was quantitated as ratio of Nanoluc/Firefly luc intensity. Data are mean + SEM of triplicate readings of three experiments for each cell line. Statistical significance was assessed by Student’s t-test (*** *P* < 0.0001)

Comparison between the WT and mutant sequence of *SLC4A4* revealed that *206G>A likely creates a Class I ARE motif (Fig. 3) that is normally lacking in *SLC4A4* 3′UTR. To examine the effect of the created ARE motif on the transcript levels, we performed RT-QPCR in LCL generated from the two-available patients’ blood and from gender-matched control. As shown in Fig. 2, this revealed marked reduction of *SLC4A4* transcript (85%, paired Student’s t-test, *P* < 0.01), which suggests that the creation of a neo-ARE motif markedly reduced *SLC4A4* mRNA levels.

### Neo-ARE in *SLC4A4* induces post-transcriptional effects in gene expression

The neo-ARE introduced in the patient’s *SLC4A4* gene with isolated ocular phenotype belongs to canonical class I ARE that harbors UUAUUUAUU in U-rich context. We first introduced the AU-rich region that contains the *206G>A mutation or the WT sequence with their flanking region in the 3′UTR of a post-transcriptional reporter vector (Fig. 3a) [26]. This selective post-transcriptional assessment system has been established with non-transcriptional inducible promoter (RPS30) and UU/UA reduced reporter coding region [26, 31]. We compared the mutant with WT 3′UTR constructs and found a consistent and appreciable reduction in the activity of the reporter fused to the ARE-forming (mutant) 3′UTR (Fig. 3b). This effect was observed in multiple different cell lines representing both of epithelial and fibroblast tissues. Specifically, there was a significant reduction (26–57%, *P* < 0.0001) depending on the cell line used (Fig. 3b).

**Fig. 4:**
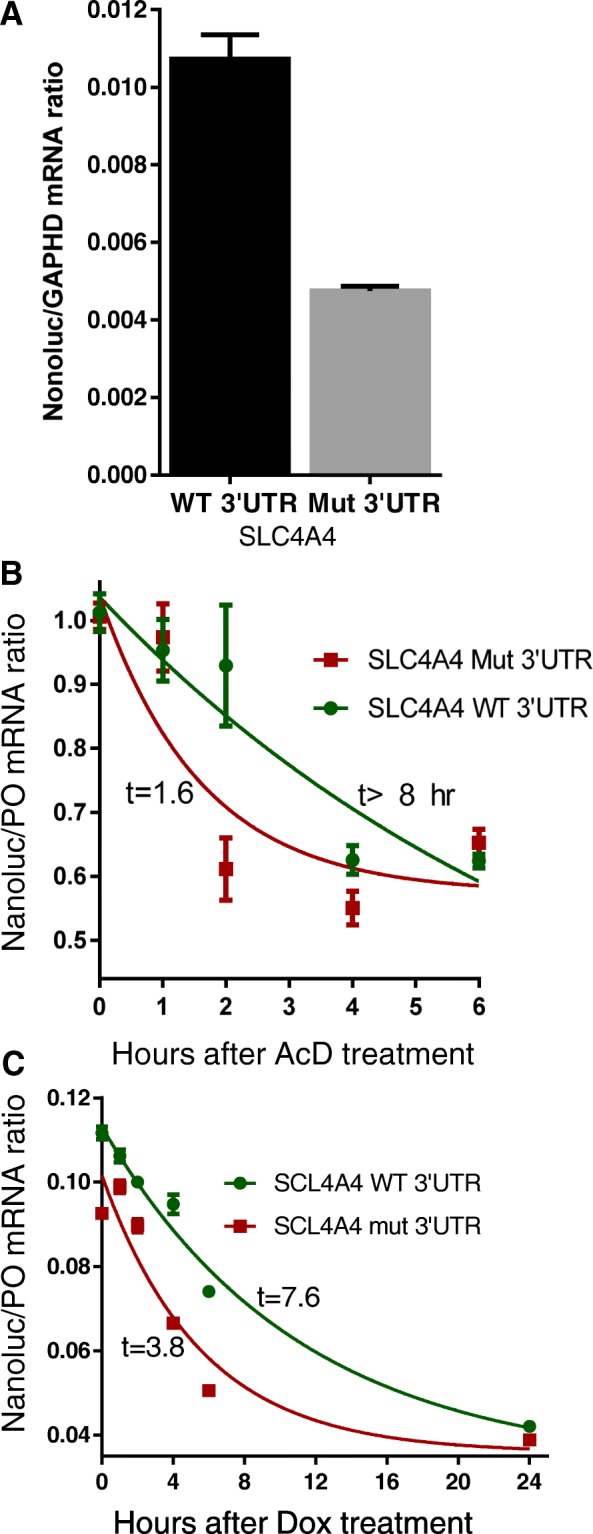
Effect of *SCL4A4* ARE-forming mutation on mRNA decay. **a** Hap-1 fibroblast-like cells were transfected with nanoluciferase reporters fused with WT *SLC4A4* 3′UTR or the ARE-forming mutant -*SLC4A4* 3′UTR for 16 h. Total RNA was extracted and levels of reporter mRNA were quantitated by RT-QPCR using TaqMan primers specific to nanoluciferase and were normalized to the housekeeping gene, *GAPDH*. Data are mRNA ratio, mean ± SEM of triplicate measurements from two experiments. Statistical significance was assessed by Student’s t-test; ***P < 0.0001. **b** Hap-1 cells were transfected with either WT or mutant *SLC4A4* 3′UTR constructs. The cells were treated with actinomycin D (5 μg/mL) up to 6 h. RT-QPCR was performed on all samples and the relative abundance level of the reporter’s transcript was taken as a measure of the ratio between the construct and an endogenous gene (RPLPO). **c** HeLa Tet-off cells (3 × 10^4^ cells/well) were transfected with TetO-linked reporters fused with WT *SLC4A4* 3′UTR or the ARE-forming mutant *SLC4A4* 3′UTRs. After 16 h, the transcription was blocked by doxycycline (1 μg/mL) for the indicated periods of time. Total RNA was extracted and subjected RT-QPCR using TaqMan primers specific to Nanouciferase mRNA. The data are presented as luciferase mRNA/RPLPO mRNA levels, mean ± SEM of replicate from one representative experiment with three replicates of at least two experiments. The mRNA half-decay calculations were performed as described in “Methods” using the one-phase exponential decay model

Using one of these cell lines for further studies, the HAP-1 fibroblast-like cell line, the mRNA levels generated from the reporter construct that was fused with 3′UTR containing the ARE-creating mutation were significantly reduced (by 60%, *P* < 0.001; Fig. 4a). Since AREs usually promote mRNA instability, we measured the mRNA half-life using two different methods and cell lines. The actinomycin D chase experiment in the HAP-1 cell line demonstrated that indeed the neo-ARE led to shorter (labile) half-life (Fig. 4b). Using Tet-off Hela, we also found the neo-ARE created by the mutation caused mRNA destabilization and significant reduction in the half-life by at least twofold (Fig. 4c).

### The *SCL4A4* neo-ARE physically associates and responds to ZFP36

**Fig. 5:**
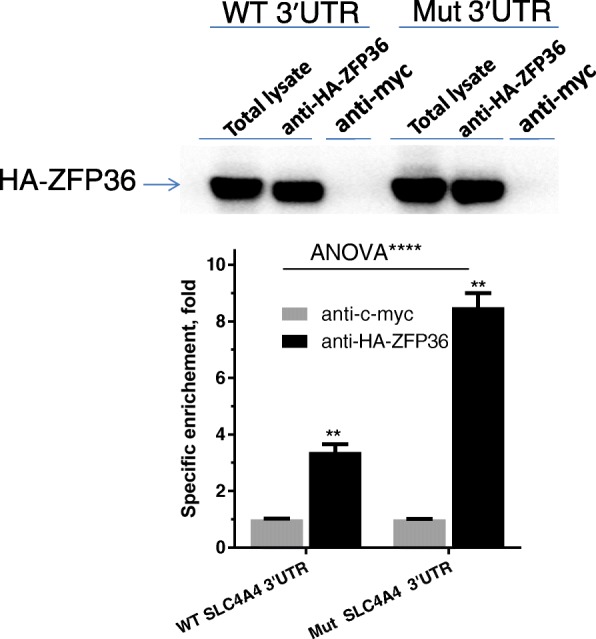
RNA-IP for *SLC4A4* ARE-forming mutation. HEK 293 cells were co-transfected with HA-ZFP36 expression vector along with either WT *SLC4A4* 3'UTR or Mut *SLC4A4* 3'UTR. Following transfection, cells were lysed and incubated with either anti-HA-ZFP36 or anti-Myc antibodies. The pulled down RBP-mRNA immune-complex with either anti-HA-ZFP or the control anti-Myc complexes were processed for immunoblotting (*upper panel*) ﻿or for cDNA RT-QPCR (*lower panel*l). First lane in the blot corresponding to total lysate was not subjected to immunoprecipitation. The mRNA levels were normalized to RPLPO as background control. Specific enrichment was calculated by reporter mRNA levels/background (RPLPO) mRNA levels and further normalized to negative control (anti-Myc) as 1.0. Data are from one representative experiment with two RT-QPCR reactions (each in triplicate). Data are mean ± SEM. Statistical significance was assessed by two-way ANOVA and Student’s t-test; **P < 0.001, ***P < 0.0001 as indicated

Because the ARE-creating mutation harbors a binding site (UUAUUUAUU) for the mRNA decay-promoting protein, ZFP36, we performed RNA-IP experiments. The HA-tagged ZFP36 (HA-ZFP36) construct was over-expressed in HEK-293 cells, followed by immunoprecipitation of HA-ZFP36 by anti-HA antibody (as confirmed by western blotting) (Fig. 5, *upper panel*). An IgG background control (anti-Myc) showed lack of immunoprecipitation of the HA-ZFP36 confirming IP specificity (Fig. 5, *upper panel*). RNA-IP clearly shows that *SLC4A4* ARE-forming mutant reporter mRNA is significantly bound to HA-ZFP36 (at least by twofold, *P* < 0.01) when compared with WT 3′UTR-fused reporter (Fig. 5, *lower panel*).

**Fig. 6:**
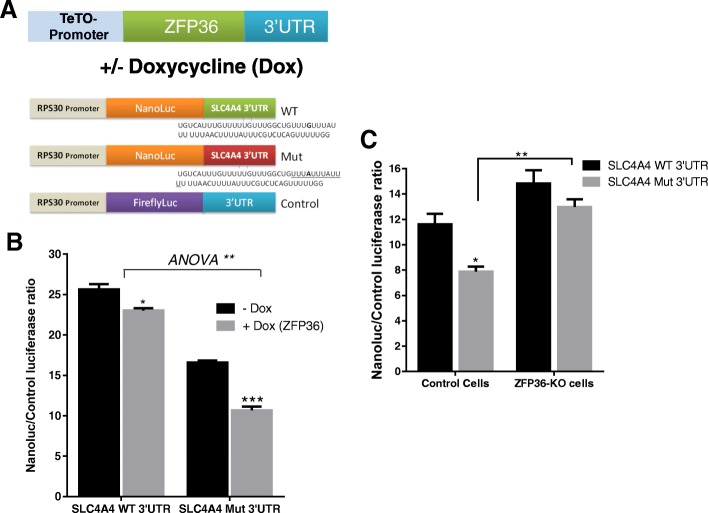
ZFP36 regulation of *SCL4A4* WT and ARE-forming mutant (Mut) reporters. **a** A scheme for the constructs and co-transfection experiments. **b** The *SCL4A4* WT or the Mut 3′UTR containing Nanoluc reporters along with Firefly control plasmid were co-transfected with a Tet-O–inducible ZFP36 expression cassette (constructed as described in “Methods”) in HEK-Tet-ON cell for 16 h. ZFP36 was induced by adding 250 ng/mL of tetracycline analog, doxycycline, for additional 16 h. **c** Parental Hap-1 fibroblast-like cells and ZFP36 CRISPR-deleted Hap-1 cells were transfected with nanoluciferase reporters fused with WT *SLC4A4* 3′UTR or the Mut -*SLC4A4* 3′UTR along with firefly luciferase normalization vector, for 16 h. Nanoluciferase activity was measured and expressed as Nanoluc/firefly luc ratio, mean ± SEM. Statistical significance was assessed by two-way ANOVA and Student’s t-test; *P < 0.05, **P < 0.01, ***P < 0.0001 as indicated. Data are mean ± SEM of triplicate measurements of at least two independent experiments

Next, we demonstrated the functional response to the neo-ARE-forming mutation to ZFP36. Tet-On ZFP36 constructs were transfected into HEK293 Tet-On cells and co-transfected with nano-luciferase reporter constructs fused with 3′UTR containing either SCL4A4 WT sequence or the ARE-forming mutation (Fig. 6a). A control non-ARE firefly luciferase construct was also co-transfected used for normalization. Induction with the tetracycline analog, doxycycline, caused a significant decrease in the expression of the reporter fused with 3′UTR of the ARE-forming mutation (35% reduction, *P* = 0.0003) when compared with the WT 3′UTR linked reporter (10% reduction, *P* = 0.02; Fig. 6b). Nearly similar results were obtained with ZFP36 CRISPR deleted HAP-1 cells (Fig. 6c). Taken together, these results show that *SLC4A4* neo-ARE created due to the hereditary mutation binds and responds to ZFP36. They also show that the ARE-creating mutation is functionally responsive to the mRNA decay-promoting protein, ZFP36.

## Discussion

AU-rich elements are among the most important regulatory elements that post-transcriptionally control the expression of many genes in mammalian cells. AREs are characterized by the presence of consensus pentamer AUUUA in U-rich context such as the nonamer UUAUUUA(U/A)(U/A) and they can exist in clusters of two to six [32, 33]. AREs are enriched in transiently expressed genes in which they play a key role in their tight temporal regulation. This control is mediated by trans-acting factors in the form of RNA-binding proteins as well as microRNAs; such effects are not limited to RNA stability regulation but also translational efficiency [34].

Despite the established role of AREs, most of the knowledge about its role in human diseases is in the context of complex diseases such as cancer rather than Mendelian diseases [35]. Indeed, we are not aware of variants creating a typical ARE (Class I or Class II) in the context of any human disease. In the families we present, we show that an isolated ocular phenotype in the form of band keratopathy with or without congenital glaucoma is linked to a novel variant that creates a neo-ARE in a gene that normally lacks it. Our data strongly suggest that this neo-ARE induces ZFP36-mediated effect on the post-transcriptional control of *SCL4A4*. The consistent lack of any syndromic feature in all patients with this mutation is in striking contrast to the known syndromic phenotype associated with coding mutations in *SLC4A4*, i.e. severe renal and ocular involvement with intellectual disability [36]. This suggests that the destabilization of *SLC4A4* mRNA was only severe enough to induce the ocular sub-phenotype of an otherwise multisystem syndrome.

Coding versus enhancer mutations are well-known to explain some instances of variable expressivity. For example, coding mutations in *SOX9* are established causes of the lethal disorder campomelic dysplasia. However, point mutations in the craniofacial-specific enhancer cause a much-limited Pierre-Robin phenotype [37]. Our finding that the creation of a neo-ARE with a tissue-specific pathological consequence, therefore, suggests a novel biological aspect of ARE that has potentially important implications for the phenomenon of variable expressivity akin to that of coding versus enhancer mutations. Enhancer elements are shared by all tissues but display a tissue-specific activity that is mediated by in *trans* factors. It is tempting to speculate that the same neo-ARE that is shared by all cells in our patients similarly exerts a tissue specific destabilizing effect via yet unidentified tissue-specific in *trans* factors. Although ZFP36, which we show mediates at least part of the destabilizing effect created by the neo-ARE, does not appear to be tissue-specific in distribution according to published data, we do not rule out the possibility that it may display variable abundance in different tissues. Alternatively, a cornea-specific non-coding RNA is another possible culprit since tissue specificity of many micro-RNA species is well documented. Finally, it is also possible that previously reported coding mutations in *SCL4A11* result in dysfunctional proteins whereas our neo-ARE mutation only affects abundance with resulting tissue-specific threshold-dependent phenotype. This would be consistent with the fact that the 15 previously reported *SLC4A4* mutations (11 missense mutations, two stopgain, and two deletion mutations) always manifested clinically in a syndromic fashion comprising renal tubular acidosis and ocular abnormalities as well as varying intellectual and growth abnormalities. Consistent with our hypothesis, none presented with isolated phenotypes.

## Conclusions

In conclusion, we report the first point ARE-creating mutation to cause a Mendelian phenotype. The creation of a neo-ARE as a result and the tissue-specific phenotypic expression of an otherwise pleiotropic disease gene suggests a novel mechanism for variable expressivity of human diseases. Investigating the role of ARE in the spatial, in addition to its established role in temporal, control of transiently expressed genes will be an important future direction.

## References

[1] Wang ET, Sandberg R, Luo S, Khrebtukova I, Zhang L, Mayr C (2008). Alternative isoform regulation in human tissue transcriptomes. Nature.

[2] Stern CO (1960). Vogt and the terms “Penetrance” and “Expressivity”. Am J Hum Genet.

[3] Ahluwalia JK, Hariharan M, Bargaje R, Pillai B, Brahmachari V (2009). Incomplete penetrance and variable expressivity: is there a microRNA connection?. Bioessays.

[4] Clark TA, Schweitzer AC, Chen TX, Staples MK, Lu G, Wang H (2007). Discovery of tissue-specific exons using comprehensive human exon microarrays. Genome Biol.

[5] Pretorius PR, Aldahmesh MA, Alkuraya FS, Sheffield VC, Slusarski DC (2011). Functional analysis of BBS3 A89V that results in non-syndromic retinal degeneration. Hum Mol Genet.

[6] Shaheen R, Rahbeeni Z, Alhashem A, Faqeih E, Zhao Q, Xiong Y (2014). Neu-Laxova syndrome, an inborn error of serine metabolism, is caused by mutations in PHGDH. Am J Hum Genet.

[7] El-Hattab AW, Shaheen R, Hertecant J, Galadari HI, Albaqawi BS, Nabil A (2016). On the phenotypic spectrum of serine biosynthesis defects. J Inherit Metab Dis.

[8] Weedon MN, Cebola I, Patch A-M, Flanagan SE, De Franco E, Caswell R (2014). Recessive mutations in a distal PTF1A enhancer cause isolated pancreatic agenesis. Nat Genet.

[9] Al‐Shammari M, Al‐Husain M, Al‐Kharfy T, Alkuraya FS (2011). A novel PTF1A mutation in a patient with severe pancreatic and cerebellar involvement. Clin Genet.

[10] Bale AE (1997). Variable expressivity of patched mutations in flies and humans. Am J Hum Genet.

[11] Shaheen R, Szymanska K, Basu B, Patel N, Ewida N, Faqeih E (2016). Characterizing the morbid genome of ciliopathies. Genome Biol.

[12] Mazumder B, Seshadri V, Fox PL (2003). Translational control by the 3′-UTR: the ends specify the means. Trends Biochem Sci.

[13] Cho T-J, Lee K-E, Lee S-K, Song SJ, Kim KJ, Jeon D (2012). A single recurrent mutation in the 5′-UTR of IFITM5 causes osteogenesis imperfecta type V. Am J Hum Genet.

[14] Pippucci T, Savoia A, Perrotta S, Pujol-Moix N, Noris P, Castegnaro G (2011). Mutations in the 5′ UTR of ANKRD26, the ankirin repeat domain 26 gene, cause an autosomal-dominant form of inherited thrombocytopenia, THC2. Am J Hum Genet.

[15] Züchner S, Wang G, Tran-Viet K-N, Nance MA, Gaskell PC, Vance JM (2006). Mutations in the novel mitochondrial protein REEP1 cause hereditary spastic paraplegia type 31. Am J Hum Genet.

[16] Dusl M, Senderek J, Müller JS, Vogel JG, Pertl A, Stucka R (2015). A 3′-UTR mutation creates a microRNA target site in the GFPT1 gene of patients with congenital myasthenic syndrome. Hum Mol Genet.

[17] Suhl JA, Muddashetty RS, Anderson BR, Ifrim MF, Visootsak J, Bassell GJ (2015). A 3′ untranslated region variant in FMR1 eliminates neuronal activity-dependent translation of FMRP by disrupting binding of the RNA-binding protein HuR. Proc Natl Acad Sci U S A.

[18] Tomaselli PJ, Rossor AM, Horga A, Jaunmuktane Z, Carr A, Saveri P (2017). Mutations in noncoding regions of GJB1 are a major cause of X-linked CMT. Neurology.

[19] Hitti E, Khabar KS (2012). Sequence variations affecting AU-rich element function and disease. Front Biosci (Landmark Ed).

[20] Cardo LF, Coto E, de Mena L, Ribacoba R, Lorenzo-Betancor O, Pastor P (2012). A search for SNCA 3′ UTR variants identified SNP rs356165 as a determinant of disease risk and onset age in Parkinson’s disease. J Mol Neurosci.

[21] Chatzikyriakidou A, Voulgari PV, Georgiou I, Drosos AA (2010). A polymorphism in the 3’-UTR of interleukin-1 receptor-associated kinase (IRAK1), a target gene of miR-146a, is associated with rheumatoid arthritis susceptibility. Joint Bone Spine.

[22] Di Paola R, Frittitta L, Miscio G, Bozzali M, Baratta R, Centra M (2002). A variation in 3′ UTR of hPTP1B increases specific gene expression and associates with insulin resistance. Am J Hum Genet.

[23] Carr IM, Flintoff KJ, Taylor GR, Markham AF, Bonthron DT (2006). Interactive visual analysis of SNP data for rapid autozygosity mapping in consanguineous families. Hum Mutat.

[24] Alkuraya FS. Discovery of rare homozygous mutations from studies of consanguineous pedigrees. Curr Protoc Hum Genet. 2012;6:12. 11-16.12. 13.1. 10.1002/0471142905.hg0612s75 23074070

[25] Alkuraya FS (2016). Discovery of mutations for Mendelian disorders. Hum Genet.

[26] Hitti E, Al-Yahya S, Al-Saif M, Mohideen P, Mahmoud L, Polyak SJ (2010). A versatile ribosomal protein promoter-based reporter system for selective assessment of RNA stability and post-transcriptional control. RNA.

[27] Al-Haj L, Al-Ahmadi W, Al-Saif M, Demirkaya O, Khabar KS (2009). Cloning-free regulated monitoring of reporter and gene expression. BMC Mol Biol.

[28] Hitti E, Bakheet T, Al-Souhibani N, Moghrabi W, Al-Yahya S, Al-Ghamdi M (2016). Systematic analysis of AU-rich element expression in cancer reveals common functional clusters regulated by key RNA-binding proteins. Cancer Res.

[29] Al-Souhibani N, Al-Ghamdi M, Al-Ahmadi W, Khabar KS (2014). Posttranscriptional control of the chemokine receptor CXCR4 expression in cancer cells. Carcinogenesis.

[30] Lek M, Karczewski KJ, Minikel EV, Samocha KE, Banks E, Fennell T (2016). Analysis of protein-coding genetic variation in 60,706 humans. Nature.

[31] Al-Saif M, Khabar KS (2012). UU/UA dinucleotide frequency reduction in coding regions results in increased mRNA stability and protein expression. Mol Ther.

[32] Zubiaga AM, Belasco JG, Greenberg ME (1995). The nonamer UUAUUUAUU is the key AU-rich sequence motif that mediates mRNA degradation. Mol Cell Biol.

[33] Bakheet T, Williams BR, Khabar KS (2006). ARED 3.0: the large and diverse AU-rich transcriptome. Nucleic Acids Res.

[34] Asirvatham AJ, Gregorie CJ, Hu Z, Magner WJ, Tomasi TB (2008). MicroRNA targets in immune genes and the Dicer/Argonaute and ARE machinery components. Mol Immunol.

[35] Khabar KS (2017). Hallmarks of cancer and AU-rich elements. Wiley Interdiscip Rev RNA.

[36] Igarashi T, Inatomi J, Sekine T, Cha SH, Kanai Y, Kunimi M (1999). Mutations in SLC4A4 cause permanent isolated proximal renal tubular acidosis with ocular abnormalities. Nat Genet.

[37] Benko S, Fantes JA, Amiel J, Kleinjan D-J, Thomas S, Ramsay J (2009). Highly conserved non-coding elements on either side of SOX9 associated with Pierre Robin sequence. Nat Genet.

